# Deep Learning to Distinguish *ABCA4*-Related Stargardt Disease from *PRPH2*-Related Pseudo-Stargardt Pattern Dystrophy

**DOI:** 10.3390/jcm10245742

**Published:** 2021-12-08

**Authors:** Alexandra Miere, Olivia Zambrowski, Arthur Kessler, Carl-Joe Mehanna, Carlotta Pallone, Daniel Seknazi, Paul Denys, Francesca Amoroso, Eric Petit, Eric H. Souied

**Affiliations:** 1 Department of Ophthalmology, Centre Hospitalier Intercommunal de Créteil, University Paris-Est Créteil, 94000 Créteil, France; oliviazambro@yahoo.fr (O.Z.); carljoe.mehanna@gmail.com (C.-J.M.); carlotta.pallone@gmail.com (C.P.); daniel.seknazi@gmail.com (D.S.); paul.denys92@gmail.com (P.D.); amorosofrancesca123@gmail.com (F.A.); eric.souied@chicreteil.fr (E.H.S.); 2 EPISEN—ISBS, University Paris-Est Créteil, 94000 Créteil, France; arthurkessler92@gmail.com; 3 Laboratory of Images, Signals and Intelligent Systems (LISSI, EA N° 3956), University Paris-Est Créteil, 94400 Vitry-sur-Seine, France; petit@u-pec.fr

**Keywords:** inherited retinal diseases, deep learning, fundus autofluorescence, retinal imaging

## Abstract

(1) Background: Recessive Stargardt disease (STGD1) and multifocal pattern dystrophy simulating Stargardt disease (“pseudo-Stargardt pattern dystrophy”, PSPD) share phenotypic similitudes, leading to a difficult clinical diagnosis. Our aim was to assess whether a deep learning classifier pretrained on fundus autofluorescence (FAF) images can assist in distinguishing *ABCA4*-related STGD1 from the *PRPH2/RDS*-related PSPD and to compare the performance with that of retinal specialists. (2) Methods: We trained a convolutional neural network (CNN) using 729 FAF images from normal patients or patients with inherited retinal diseases (IRDs). Transfer learning was then used to update the weights of a ResNet50V2 used to classify the 370 FAF images into STGD1 and PSPD. Retina specialists evaluated the same dataset. The performance of the CNN and that of retina specialists were compared in terms of accuracy, sensitivity, and precision. (3) Results: The CNN accuracy on the test dataset of 111 images was 0.882. The AUROC was 0.890, the precision was 0.883 and the sensitivity was 0.883. The accuracy for retina experts averaged 0.816, whereas for retina fellows it averaged 0.724. (4) Conclusions: This proof-of-concept study demonstrates that, even with small databases, a pretrained CNN is able to distinguish between STGD1 and PSPD with good accuracy.

## 1. Introduction

The adenosine triphosphate-binding cassette, subfamily A, member 4 (*ABCA4*) gene encodes for a membrane-associated protein located in the outer segment (OS) disc membranes of rod and cone photoreceptors [1,2]. Mutations in the *ABCA4* gene are a known cause for recessive Stargardt disease (STGD1). STGD1 follows an autosomal recessive pattern of inheritance, with usual disease onset in the second decade of life. Nevertheless, albeit rare, late-onset forms of STGD1 do exist [3,4]. The characteristic fundus features of STDG1 disease include irregular yellow-white fundus flecks, atrophic macular lesions, and sparing of the peripapillary area by both flecks and atrophy [4,5,6].

Despite this, the association of these findings is considered pathognomonic to STGD1; the phenotype is not necessarily exclusive to STGD1. Multifocal pattern dystrophy simulating STGD1 (“pseudo-Stargardt pattern dystrophy”) is an autosomal-dominant inherited retinal disease, caused by a *PRPH2/RDS* mutation that may simulate STGD1 [7]. Pseudo-Stargardt pattern dystrophy (PSPD) patients may display yellowish fundus flecks and chorioretinal atrophy [7].

Fundus autofluorescence (FAF) is an in vivo imaging method for the metabolic mapping of lipofuscin accumulation in the retina [8,9,10]. Studies on animal models, as well as histopathological studies, have demonstrated that the formation of retinal pigment epithelium (RPE) cell lipofuscin is augmented and pathogenic in *ABCA4*-associated disease [5,10,11,12]. Moreover, quantitative autofluorescence (qFAF) studies have shown that high levels of qFAF are a hallmark of *ABCA4*-positive patients, but the qFAF levels were also elevated in eyes with a *PRPH2/RDS* mutation [13]. Therefore, in both STDG1 and PSPD, FAF reveals a similar phenotype.

Despite the overlap between STGD1 and PSPD, the natural history is different, with only mild vision loss until the advanced stages of the disease for PSPD patients, and hence a better prognosis than STGD1 patients, making a correct diagnosis essential in these patients.

Deep learning approaches require large volumes of high-quality training data, which may be difficult in the case of rare inherited retinal diseases having undergone genetic screening. Moreover, the pretrained networks used in the current literature undergo transfer learning using the ImageNet dataset, containing color photography. Thus, our objective was to consider whether a deep learning classifier, pretrained using various FAF images, can assist in distinguishing *ABCA4*-related STGD1 from PSPD caused by mutations in *PRPH2/RDS,* and to compare these findings with the grading of retinal specialists.

## 2. Materials and Methods

### 2.1. Image Database

Patients retrospectively included in this study had (1) a confirmed *ABCA4* mutation and associated STGD1 at various stages of progression OR (2) a confirmed *PRPH2/RDS*-associated disease with a PSPD phenotype. Genetic diagnosis was established by high-speed sequencing of genes involved in hereditary diseases on HiSe. Of note, only 1 pathogenic *ABCA4* variant was required for STGD1 patients to be included. This retrospective study was conducted in accordance with the tenets of the Declaration of Helsinki. Written consent was waived due to the retrospective nature of the study.

We used macula-centered fundus autofluorescence retinal images from genetically confirmed eyes with STGD1 or PSPD, in the Department of Ophthalmology of Créteil, France. FAF images had been obtained in the Ophthalmology outpatient clinic in the Department of Ophthalmology in Créteil between April 2007 and April 2020, using Spectralis HRA + OCT (Heidelberg Engineering, Heidelberg, Germany). High-resolution (1536 × 1536 pixels), 30° × 30°, as well as 55° × 55° field-of-view images, centered on the fovea, with a minimum averaging of 30 frames, were extracted. One FAF image/eye/year of each patient was extracted. All images were deidentified, and all personal data (e.g., patient name, birth date, and study date) were removed. FAF images were not cropped but were resized to 224 × 224 pixels to meet the network specifications, with the fovea at the center, and were labeled as either STGD1 or PSPD, according to the specific mutation obtained following genetic testing.

A two-class classification system (STGD1 and PSPD) was implemented. The images were then partitioned into three sets using the function train_test_split of scikit-learn: the training set (60% of the images), the validation set (10% of the images), and the test set (30% of the images). The assignment of the images towards the training, the validation, and the testing sets was performed randomly. The training, validation and test data were strictly separated to prevent correlations. The images of patients used for training the deep learning classifier were not used to test it.

### 2.2. Development of a Deep Learning Classifier

**Figure 1:**
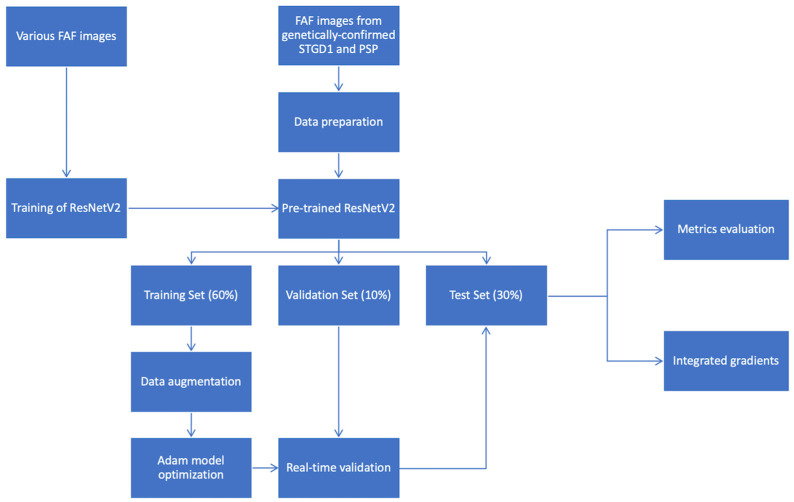
Illustration of the development of the deep learning model used. Various fundus autofluorescence images were extracted from the Créteil database and were used to train ResNet50V2. The pretrained network was used to classify genetically confirmed STGD1 and PSPD FAF images. The images were randomly partitioned into three sets: the training set (60% of the images), the validation set (10% of the images), and the test set (30% of the images). Data augmentation was performed on the training set to increase the original dataset and to reduce overfitting of the final model. The model was optimized using the Adam Optimization Algorithm. The model was then evaluated with the test set of 111 images. The output of the model was the metric evaluation of the performance of the model (accuracy, sensitivity, specificity, precision, recall, F1-score) and integrated gradient visualization.

We used ResNet50V2 to perform the classification task. We pretrained the ResNet50V2 model using 729 FAF images from patients with either normal FAF (73 images) or IRDs without genetic confirmation (656 images). The loss function used was cross-entropy. The model was optimized using the Adam Optimization Algorithm [14]. The Reduce Learning Rate on plateau was applied in order to allow the optimizer to more efficiently find the minimum in the loss surface. The model was then evaluated with the test set of 111 images (Table 1). By using integrated gradients, attribution maps were generated, allowing the impact of each pixel in the classification to be assessed, and showing which areas the model relies on to perform the classification [15]. The method is summarized in Figure 1. The performance was assessed through a comparison of the CNN’s output to the ground truth, which was set by genetic confirmation of either STGD1 (*ABCA4* mutation) or PSPD (*PRPH2/RDS* mutation).

### 2.3. Evaluation of Retina Specialists’ Performance

Graders evaluated the FAF images to distinguish between STDG1 and PSPD based on typical features. For STGD1, typical features were considered to be the presence of hyperautofluorescent flecks, presence/absence of hypoautofluorescent areas of atrophy, and presence of peripapillary sparing. For PSPD, the typical features were considered to be central hypoautofluorescent lesion with jagged border, butterfly-shaped hyperautofluorescent lesions in the macula, and absence/presence of peripapillary sparing [7,8,13].

The graders were masked to the mutation, to the grades from previous retinal imaging, and to all clinical data. Four graders (2 senior retina specialists: E.S. and O.Z., and 2 retina fellows: D.S. and P.D.) evaluated each FAF image independently. The same metrics (accuracy, sensitivity, specificity) were computed for human grading. The expert grader’s performance was compared to the deep learning classifier’s performance.

## 3. Results

### 3.1. Deep Learning Classifier

In this study, we included 304 FAF images from 80 eyes of 40 patients with genetically confirmed STGD1 (mean age, 47.20 ± 18.73 years) and 66 images from 18 eyes of nine patients with PSPD (mean age, 51.22 ± 12.81 years). Table 2 summarizes their demographic and genetic data.

Using a pretrained classifier on FAF images, we obtained an overall accuracy of 0.882 on the test dataset of 111 images. The AUROC was 0.89. The test loss was 0.413. The precision was 0.883. The recall (sensitivity) was 0.883. The F1-score was 0.884. Of the 91 STGD1 FAF images, 88 were correctly classified. The sensitivity for STGD1 was 0.967, while the specificity was 0.50. The positive predictive value was 0.897 and the negative predictive value was 0.769 for STGD1.

Of the 20 PSPD FAF images, 10 were correctly classified. Conversely, the sensitivity for PSPD was 0.5, with a specificity of 0.967. The positive predictive value was 0.769 and the negative predictive value was 0.897 for PSPD. The results on the training, validation, and test sets are presented in Table 3.

**Figure 2:**
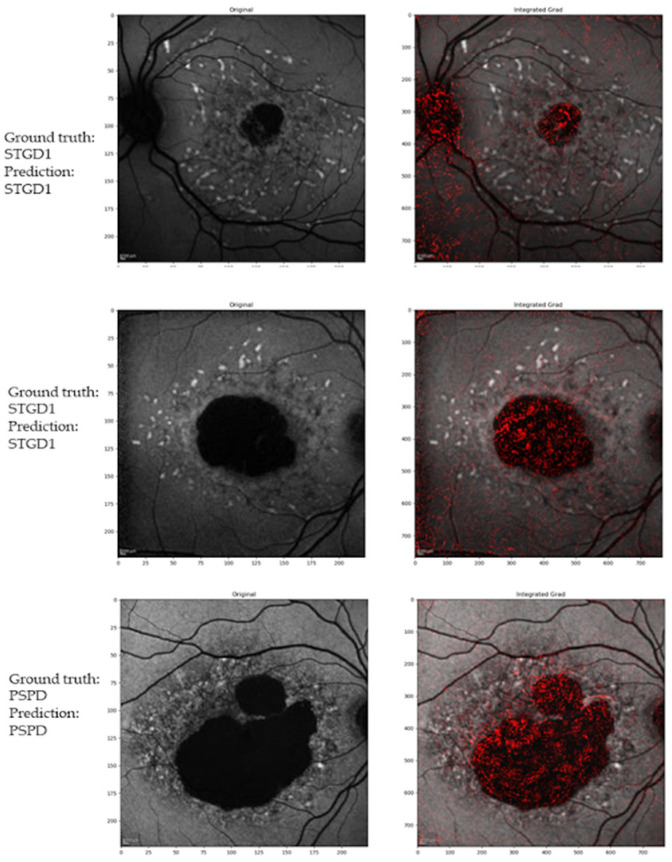
Example of correct attribution and integrated gradient visualization. Upper and middle panels: Stargardt disease (STGD1) correctly classified. Lower panels: Pseudo-Stargardt Pattern Dystrophy (PSPD) correctly classified.

**Figure 3:**
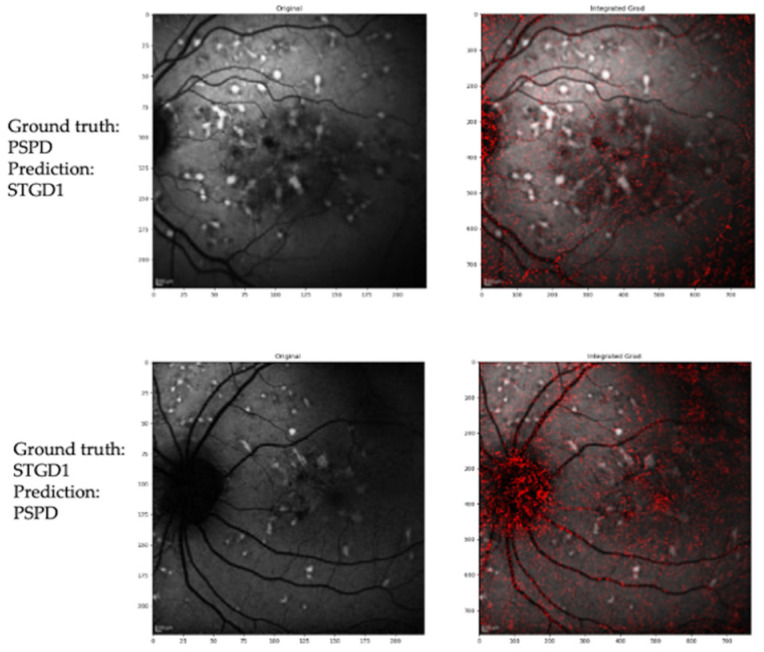
Example of incorrect attribution and integrated gradient visualization. Upper panels: Pseudo-Stargardt Pattern Dystrophy (PSPD), classified as Stargardt disease by the CNN model. Lower panels: Pseudo-Stargardt Pattern Dystrophy (PSPD), classified as Stargardt disease by the CNN model.

Figure 2 illustrates examples of correct prediction of the DL model, while Figure 3 shows examples of an incorrect prediction.

### 3.2. Evaluation of Retina Specialists’ Performance

The retina specialists consisted of two retina experts (E.S. and O.Z.) and two retina fellows (D.S. and P.D.). The diagnostic accuracy for retina experts averaged 0.816, whereas for retina fellows it averaged 0.724. The sensitivity for the detection of STGD1 on FAF imaging was higher than for PSPD detection, both for retina experts and for retinal fellows (for retina experts: 0.828 versus 0.777; for retina fellows: 0.828 versus 0.363). In terms of specificity, it was higher for PSPD compared to STGD1 for both retina experts and for retinal fellows. The interclass correlation (ICC) between the four human graders was 0.242. The evaluation of retina specialists’ performances in distinguishing, using FAF images, STGD1 from PSPD, is summarized in Table 4.

## 4. Discussion

In this study, we evaluated the performance of a deep learning classifier in distinguishing between STGD1 and PSPD on FAF, and we compared the results with those of retina specialists with varying levels of expertise. The ground truth consisted of STGD1 and PSPD with a genetic molecular diagnosis. The human graders were masked to the mutation, to the grades from previous retinal imaging, and to all clinical data.

The accuracy of the DL model was non-inferior to that of the retina experts (accuracy: 0.88 versus 0.816), as shown in Table 3 and Table 4. Regarding the DL model’s performance, on the one hand, the training loss in Table 3 indicated how well the model fitted the images in the training dataset. On the other hand, the validation loss indicated how well the model fitted new data from the validation dataset. Differences between training and validation loss may be signs that the model is overfitting or underfitting, but are also dependent on model regularization, and may also be due to the difficulty of the images in the respective datasets, and their proportion. In our model, we used 60% of the data to train the model and only 10% to validate the model, which may explain the difference in the training and validation loss. However, when applied to the test dataset containing 30% of images, the test loss was very close to the training loss, suggesting the rather good generalization capacity of the model. Moreover, the accuracy of the model was superior to the accuracy of retina fellows (relying solely on FAF images), who had an accuracy of 0.724 (Table 4). Although there was a lower overall accuracy of the retina specialists, the sensitivity and specificity for each class were more consistent compared to the same metrics of the DL model. The higher accuracy of the DL model was derived from the higher recall/sensitivity compared to human readers (0.883 versus 0.790 for retina experts and 0.595 for retina fellows). Nevertheless, the class imbalance (304 STGD1 FAF images and 66 PSPD images) was reflected by poor sensitivity results for PSPD by the DL model. Indeed, with only 10 out of 10 PSPD images correctly classified by the model in the test set, despite the model’s overall good accuracy, the proportion of PSPD that were correctly identified was 50%. Moreover, a poor specificity was found for STGD1, showing that the proportion of true negatives that were correctly identified was low, and that of false positives high, due to the misclassification of PSPD as STGD1.

There are several reasons for these low values of sensitivity and specificity. Both STGD1 and PSPD have a phenotypic similarity in multimodal imaging, including FAF. While the model correctly relies on pixels within the area of atrophy and flecks, the similarity between the two images, and the inconsistency of differentiating features (i.e., peripapillary sparing), may be misleading for the DL model, as for the clinician. An important point is that, on 30° × 30° FAF images, the presence of the optic disc and the subsequent (when present) peripapillary sparing was inconstant, which may help further explain the model’s performance.

Furthermore, the class imbalance may also have contributed to the difference between the specificity and sensitivity of each class. Moreover, functional testing, such as full-field ERG in PSPD, is often normal [13], but high variability is present even in families carrying the same mutation [7]. In previous studies, while neither FAF nor spectral-domain optical coherence tomography (SD-OCT) were able to distinguish between STGD1 and *PRPH2*-associated PSPD, qFAF was shown to be lower in patients with PSPD compared to STGD1 [13,16]. As genetic molecular diagnosis is not an easily carried out, and as the prognosis of (late-onset) STGD1 is different from PSPD, with further improvements and larger datasets such a DL model may assist physicians in differentiating the two diseases.

Given the inherently small databases, and in order to improve the classification accuracy, ResNet50V2 was pretrained with various FAF images to learn how to extract features specific to FAF. Despite our limited database (304 FAF images of STGD1 and 66 images of PSPD) and the class imbalance, the pretrained ResNet50V2 obtained a good accuracy of 0.88. Other approaches may have been implemented in order to deal with the small sample size. In practice, it takes hundreds of thousands of images to optimize the innumerable parameters of a CNN. We pretrained ResNet50V2 using 729 FAF images from patients with either normal FAF (73 images) or other IRDs without genetic confirmation (656 images). Pretraining the CNN with a larger sample size could have led to a better performance of the model. We performed data augmentation only on the training set within the Keras framework. Although data generation of the input database has been previously performed in a study classifying diabetic retinopathy using OCT Angiography [17], as it may generate small variations of the training dataset into the test dataset, leading to an overestimation of the DL model’s accuracy, this method was not used here.

If both age-related macular degeneration and diabetic retinopathy are retinal diseases where, due to the high incidence and prevalence, large-scale, publicly available databases exist, predominantly consisting of color fundus photography and/or OCT images, this is not true for IRDs. In IRDs, deep learning applications are developing, but are still at an early stage due to the rarity of these diseases. Our group recently focused on the applications of DL to classify IRDs using FAF, obtaining excellent accuracies when distinguishing STGD1, retinitis pigmentosa, BD and healthy controls [18], as well as to distinguish the chorioretinal atrophy of genetic or degenerative causes [19]. Furthermore, the literature has also recently focused on distinguishing STGD1 from healthy controls, using OCT as the imaging technique for the DL classification, and obtaining high accuracies despite the relatively small datasets [20]. Other groups have also focused on the prediction of causative genes in IRDs from color fundus photography, FAF imaging [21], or SD-OCT [22].

This study has several limitations, of which the main is the small dataset. Given that STGD1 and PSPD, while presenting a phenotypic overlap on FAF imaging, are consequent to mutations in different genes (*ABCA4* for STGD1 and *PRPH2* for PSPD), a genetic molecular diagnosis for all included eyes was essential. These databases would be extremely useful, even more so when dealing with rare, orphan diseases. Therefore, the ultimate and best solution for increasing the accuracy of our model would be multi-institutional collaborations. Another limitation is the fact that we included various stages of STGD1, with various degrees of overlap with *PRPH2*. To date, no reference databases of FAF imaging are available. Finally, while the image size was reduced to fit CNN requirements, the FAF images were not cropped, leading to a variability within the database with regard to the visualization of the optic disc and peripapillary sparing, which may have additionally impacted the results.

Last but not least, it is important to keep in mind that the comparison of a deep learning classifier and retina specialists to distinguish STGD1 from PSPD relies solely on the features in a single FAF image. In a clinical setting, a diagnosis by a physician is made with the consideration of other clinical parameters such as the age of the patient, time of onset, family history and a battery of other imaging and functional tests. The high number of graders, leading to a greater difficulty to reach agreement, as well as the phenotypic similarity between STGD1 and PSPD, may explain the fair agreement between the different human readers. This highlights the relevance of the CNN in detecting these phenotypically similar diseases, which present diagnostic challenges to the clinician and are solely based on FAF imaging. Larger studies with larger datasets and, ideally, a multimodal approach, are needed before implementation in a clinical setting.

## 5. Conclusions

Our results show the efficiency of training a CNN with transfer learning, generating a stable classification performance despite the small dataset. Therefore, pretraining the model with the same type of imaging may prove useful when it is difficult to amass image data, such as in cases of rare genetic diseases.

This proof-of-concept study demonstrates that a DL-based distinction between STGD1 and PSPD is possible using FAF images, with a good accuracy, sensitivity, and specificity compared to retinal experts relying solely on FAF images.

## Figures and Tables

**Table 1:** The split of the dataset for each class consisted of a training set (60%), a validation set (10%) and a test set (30%). (*n*)—number of fundus autofluorescence (FAF) images in each dataset.

	Training Set	Validation Set	Test Set	Total
Stargardt disease (*n*)	183	30	91	304
Pseudo-Stargardt Pattern Dystrophy (*n*)	40	6	20	66
Total (*n*)	223	36	111	370

**Table 2:** Demographic and genetic data.

	Patient	Age	Sex	Mutation
*RDS/PRPH2* mutation	#1	49	M	c.639c > G (p.Cys213Trp) *RHO*: c.185C > A (Thr62Asn)
	#2	50	F	c.639c > G (p.Cys213Trp)
	#3	51	F	c623G > A (p.Gly208Asp)
	#4	83	M	c.461del (p.Lys154Argfs*102)
	#5	54	M	c.461del (p.Lys154Argfs*102)
	#6	49	M	c.628C > G (p.Pro210Ala)
	#7	43	M	NA
	#8	39	F	NA
	#9	43	F	NA
*ABCA4* mutation	#1	50	M	c.3259G > A (p.Glu1087Lys) c.5882G > A (p.Gly1961Glu)
	#2	36	M	c.1749G > C (p.Lys583Asn) c.3916delinsGT (p.Pro1306Valfs*116)
	#3	36	F	c.1222C > T (p.Arg408*) c.6320G > A(p.Arg2107His)
	#4	30	M	c.2966T > C (p.Val989Ala) c.5318C > T (p.Ala1773Val)
	#5	71	M	c.1648G > A (p.Gly550Arg) c.5603A > T (p.Asn1868Ile)
	#6	14	M	c.4918C > T (p.Arg1640Trp) c.5087G > A (p.Ser1696Asn)
	#7	39	F	c.2123T > C (p.Met708Thr) c.3058dup (p.Val1020Glyfs*3)
	#8	41	F	c.3322C > T (p.Arg1108Cys) c.5885G > A (p.Gly1961Glu)
	#9	68	F	c.1015T > G (p.Trp339Gly) c.5603A > T (p.Asn1868Ile)
	#10	56	M	c.2966T > C (p.Val989Ala) c.3289A > G (p.Arg1097Gly)
	#11	25	F	c.1018T > C (p.Tyr340His) c.5315G > A (p.Trp1772*) ‡
	#12	25	F	c.5018 + 2T > C(IVS35 + 2T > C) c.5196 + 1137G > A ‡
	#13	66	F	c.4685T > C (p.Ile1562Thr) c.5113C > T (p.Arg1705Trp)
	#14	44	M	c.452T > C(p.Ile151Thr) ‡ c.3352C > T(p.His1118Tyr) ‡
	#15	71	M	c.1671T > A (p.Tyr557*) c.4139C > T (p.Pro1380Leu)
	#16	17	M	c.3813G > C (p.Glu1271Asp) ‡ c.455G > A (p.Arg152Gln) ‡ c.3322C > T (p.Arg1108Cys) ‡ c.6320G > A (p.Arg2107His) ‡
	#17	37	M	c.5363C > T (p.Pro1788Leu) c.1054G > A (p.Asp352asn) ‡, c.5882G > A (p.Gly1961Glu) ‡
	#18	45	F	c.5885G > A (p.Gly1961Glu) c.1648G > A (p.Gly550Arg) ‡, c.5603A > T (p.Asn1868Ile) ‡
	#19	17	M	c.1015T > G (p.Trp339Gly) c.2588G > C (p.Gly863Ala) c.1715G > A (p.Arg572Gln) ‡
	#20	63	F	c.5603A > T (p.Asn1868Ile) c.614G > A (p.Cys205Tyr) ‡
	#21	50	M	c.3113C > T (p.Ala1038Val) c.455G > A (p.Arg152Gln) ‡, c.3322C > T (p.Arg1108Cys) ‡, c.6320G > A (p.Arg2107His) ‡
	#22	75	F	c.455G > A (p.Arg152Gln) ‡, c.3322C > T (p.Arg1108Cys) ‡, c.6320G > A (p.Arg2107His) ‡
	#23	43	M	c.514G > A (p.Gly172Ser) ‡, c.4875T > A (p.His1625Gln) ‡, c.6094C > T (p.His2032Tyr) ‡
	#24	64	F	c.1749G > (p.Lys583Asn)
	#25	73	M	c.3916delinsGT (p.Pro1306Valfs*116)
	#26	25	M	c.1749G > C (p.Lys583Asn)
	#27	43	M	c.1749G > C (p.Lys583Asn)
	#28	87	F	c.735T > G (p.Tyr245*)
	#29	38	F	c.3813G > C (p.Glu1271Asp)
	#30	70	F	c.5363C > T (p.Pro1788Leu)
	#31	37	M	c.1749G > C (p.Lys583Asn)
	#32	67	M	c.5885G > A (p.Gly1961Glu)
	#33	68	F	c.769–784C > T (p.Leu257Aspfs*3) ‡
	#34	43	M	c.4070C > T (p.Ala1357Val)
	#35	51	F	c.1804C > T (p.Arg602Trp)
	#36	49	F	c.1804C > T (p.Arg602Trp)
	#37	25	F	c.634C > T (p.Arg212Cys)
	#38	55	M	c.5315G > A (p.Trp1772*)
	#39	20	F	c.1018T > C (p.Tyr340His)
	#40	54	M	c.1018T > C (p.Tyr340His)

NA: not available; ‡: variant.

**Table 3:** Loss, accuracy, and AUROC on the training, validation, and test sets.

		Loss	Accuracy	AUROC
ResNet50V2	Training set	0.342	0.869	0.925
	Validation set	0.6383	0.769	0.837
	Test set	0.413	0.882	0.892

**Table 4:** Retina specialists’ performances in distinguishing Stargardt disease (STGD1) from pseudo-Stargardt pattern dystrophy (PSPD) using solely the FAF imaging.

	Accuracy	Sensitivity (Recall)	Specificity
Retina expert	0.816	0.790	0.801
Retina fellow	0.724	0.595	0.590

## Data Availability

Data are available from the corresponding author upon reasonable request.
